# Evaluating the effectiveness of an innovative sleep technology to support individuals who experience sleep problems and worry-related stress: a crossover randomized controlled trial

**DOI:** 10.3389/fpsyg.2026.1912736

**Published:** 2026-09-30

**Authors:** Henriette S. Brummer, Tsjitske M. Haanstra, Luc P. De Witte

**Affiliations:** Technology for Health Care Research Group, Centre of Expertise Health Innovation, The Hague University of Applied Sciences, The Hague, Netherlands

**Keywords:** crossover design, insomnia, randomized controlled trial, sleep technology, stress

## Abstract

Insomnia and worry-related stress are common problems affecting both mental and physical health. Assistive sleep technologies such as the Somnox 2 may improve sleep quality and reduce stress. This study investigated whether the Somnox improves sleep quality and reduces perceived stress in adults with sleep problems and worry-related stress. A secondary objective was its impact on quality of life. In a 14-week crossover randomized controlled trial, 40 adults (90% female), reporting various types of worries, participated. Each completed two 6-week periods of either Somnox use or no intervention, separated by a 1-day washout. Outcomes were assessed using the Sleep Condition Indicator (SCI), Perceived Stress Scale (PSS), Mental Health Quality of Life (MHQoL), and daily self-reported sleep and stress ratings. Compared with the control condition, Somnox use was associated with higher sleep quality (SCI: +4.22 ± 5.46, 95% CI 2.48 to 5.97, *p* < 0.001) and lower perceived stress (PSS: −1.80 ± 5.61, 95% CI −3.59 to −0.01, *p* = 0.049). The SCI finding was supported by a baseline-adjusted first-period sensitivity analysis. Daily sleep scores increased (+0.15 ± 0.33, *p* = 0.012) and daily stress decreased (−0.11 ± 0.23, *p* = 0.056). No clear improvements were observed in quality of life. These findings suggest that Somnox is a feasible and promising sleep technology to improve sleep quality and possibly reduce worry-related stress in adults with worries. Future research should explore longer-term effects and diverse subgroups.

## 1 Introduction

Insomnia is a common condition that negatively affects not only those who suffer from it, but also society as a whole. Symptoms of insomnia include difficulty falling asleep, difficulty staying asleep, or waking up too early. In Netherlands, 63% of the population is unsatisfied with their sleep quality, and one in five individuals experiences some form of insomnia (CBS, 2009, 2018). Similar trends are observed in other countries, with an estimated 10%–20% of the population affected by sleep disturbances, indicating that insomnia is a global issue (Buysse, 2013; Chattu et al., 2018).

Insomnia is frequently associated with both mental and physical health problems, such as fatigue, anxiety, reduced attentional capacity, and lower work productivity. Moreover, it has a considerable impact on overall quality of life (CBS, 2018; Gardani et al., 2022; Riemann et al., 2022). Insomnia can cause individuals to experience worries and stress, which negatively influence both physical and mental health. However, the relationship is bidirectional; individuals dealing with, for example, financial difficulties or problematic home situations often experience higher levels of stress and are therefore more likely to suffer from insomnia (Hall et al., 2008; Kuo et al., 2023). This lack of sleep subsequently reinforces health complaints and decreases the individual’s vitality (Kyle et al., 2010). Vitality plays an essential role in an individual’s resilience and is reflected in medical and psychological health as well as overall life satisfaction. Consequently, a challenging vicious cycle emerges in which insomnia, reduced vitality and poor medical and psychological health mutually reinforce each other.

Two common approaches for insomnia treatment are medication and sleep technologies. More than two million people in Netherlands rely on sleep medication to improve their sleep quality (CBS, 2009). However, such medication carries disadvantages, including the risk of dependency, side effects, and often long-term use (Mitchell et al., 2012).

Somnox 2 is an assistive sleep technology developed by Somnox B.V. The device assists users by guiding their breathing patterns through sensors that detect the user’s respiratory frequency. When the user holds the device, the sensors first register the user’s breathing pace. The device then synchronizes with the user’s physical breathing rhythm and gradually slows its own rhythm, enabling the user to adjust their breathing accordingly and gradually relax. In addition to these basic settings of the device, a Somnox smartphone application is available that allows users to adjust personalized breathing speeds and rhythms. The app also enables the device to produce calming sounds, such as nature sounds or white noise, which can further enhance the relaxing effect.

This study aims to investigate whether the use of the Somnox can help people who experience a high level of stress and a poor sleep quality due to worries to improve sleep quality and reduce perceived stress. A secondary research question is what the effect of the use of the Somnox is on the quality of life of people who experience stress and sleep problems due to worries. While previous internal research by Somnox B.V. suggests potential benefits of the device for sleep quality and stress reduction, independent empirical evidence remains scarce (Somnox Onderzoek and Validatiestudie, 2023).

## 2 Materials and methods

### 2.1 Study design and procedure

This 14-week study used a crossover randomized controlled trial (RCT) design to estimate the treatment effect of the Somnox (Li et al., 2015). In this design, participants receive two study conditions (intervention and control) in a randomized order, allowing each participant to serve as their own control. The two study conditions were compared and the treatment effect was estimated by first calculating the within-individual difference for each participant, that is the difference in outcome between the two conditions for that same participant. These within-individual differences were then averaged across all participants to obtain the overall treatment effect. By comparing participants to themselves, this approach reduces variability between participants, increases statistical efficiency, and therefore requires a smaller sample size compared to a parallel-group design. Carryover effects were assumed to be minimal, and therefore a 1-day washout period was considered sufficient. The study was not prospectively registered in a clinical trial registry, as the study was considered to fall outside the scope of the Dutch Medical Research Involving Human Subjects Act (WMO).

The study procedure consisted of the following steps (Figure 1): (a) screening of participants, enrollment and random assignment into one of the two conditions, (b) 1 week of baseline measures (baseline period) with no intervention, (c) 6 weeks of either Somnox use (intervention period) or no Somnox use (control period), (d) a “washout” day, (e) 6 weeks crossover, and (f) 1 week follow-up. Outcome measures were measured at the end of the baseline period (b), at the end of the first period of 6 weeks (c), at the end of the crossover period of 6 weeks (e), and during the follow-up week (f).

**FIGURE 1:**
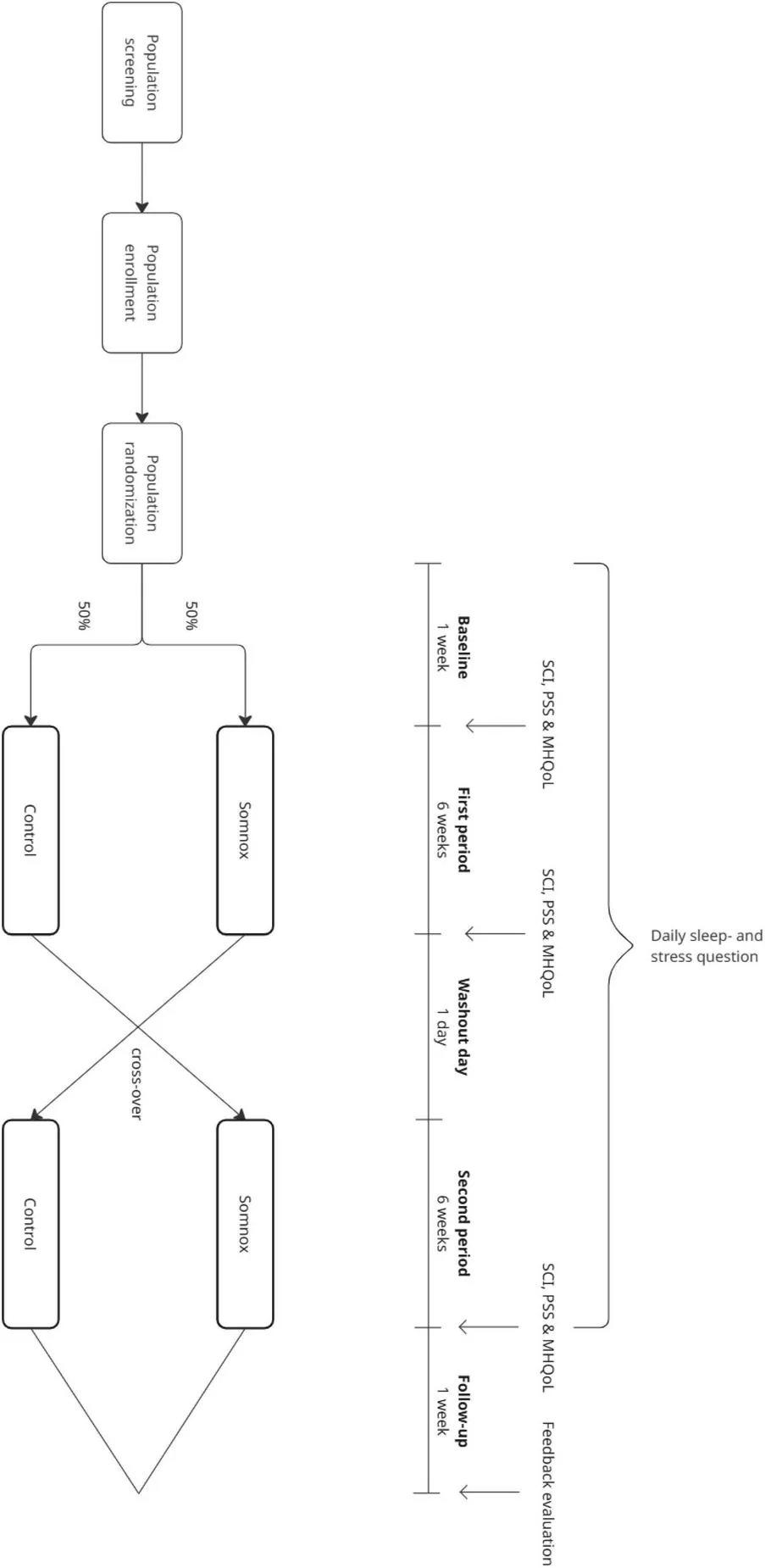
Study procedure.

### 2.2 Participants inclusion- and exclusion criteria

Participants eligible for this study included healthy adults (18+) with presence of self-reported poor sleep quality and stress due to worrying, no known current disease, and willingness and ability to comply with the study protocol. Participants excluded from this study were those that had used the Somnox in the past, had a diagnosis of a disease or condition (e.g., sleep apnea or restless legs syndrome), physical disabilities or conditions that prevented proper use of the Somnox, problematic drug use (≥3x a week use of soft-drug or any use of hard-drug), excessive alcohol use (≥14 drinks a week), known pregnancy, participation in any other study or use of a medical apparatus during the night (CPAP-apparatus, defibrillator, pacemaker). Participants were recruited in three ways. First, in-person presentations were held at partner organizations of Schuldenlab070 in The Hague, which focuses on preventing and supporting people with financial problems, to recruit potential participants who experience sleep problems and stress related to financial worries. Second, employees of The Hague University of Applied Sciences who experienced worries, stress and sleep problems were invited to participate via an online announcement on the university’s internal communication platform, through which they could respond if they were interested. Third, employees of the Haga Hospital were recruited after on-site presentations in departments where staff experience sleep difficulties and stress due to worries related to irregular work shifts. Participants were considered enrolled once they signed the informed consent and were informed that they had the right to withdraw from the study at any given time.

### 2.3 Randomization

After the participants were screened and enrolled in the study, they were sequentially randomized to one of the two conditions (intervention or control) using a 1:1 allocation ratio, with a block size of maximum 5. Enrollment was staggered, so that not all blocks of participants started the study simultaneously, allowing each block of participants to begin at their own time point.

### 2.4 Intervention and control conditions

The intervention consisted of use of the Somnox for 6 weeks. The participants received a 15-min training on how to use the Somnox prior to the intervention. The participants were instructed to use the Somnox on a daily basis, particularly during nighttime sleep, and at any preferable time, such as during daytime for relaxation, for sleep initiation or during nighttime awakenings. They had the choice to adjust the breathing and sound settings to their personal preferences. The device was used exclusively by the participant, and participants were asked to maintain their usual medication intake, sleep-wake routines and daily activities, including normal eating and drinking habits. The control condition entailed no intervention for 6 weeks.

### 2.5 Outcome measurements

The participants’ gender and the types of worries contributing to their stress and sleep problems were collected for descriptive purposes through an open-ended question. The primary outcome measure was the Sleep Condition Indicator (SCI), an 8-item questionnaire based on DSM-5 criteria for insomnia with total scores ranging from 0 to 32, where higher scores indicate better sleep quality (Espie et al., 2018). The SCI has shown good psychometric properties and discriminant validity for identifying insomnia-related symptoms (Wong et al., 2017). An SCI improvement of seven points represents reliable change (Espie et al., 2018). Secondary outcome measures included the Perceived Stress Scale (PSS) questionnaire and the Mental Health Quality of Life (MHQoL) questionnaire. The PSS assesses the perceived stress over the past month with scores ranging from 0 to 40, where higher scores indicate greater stress (Cohen et al., 1983). Previous research has demonstrated good internal consistency, test–retest reliability, and construct validity of the PSS (Lee, 2012). The MHQoL consists of the MHQoL-7D (range 0–21) and the MHQoL-VAS (range 0–10), assessing the quality of life and the psychological wellbeing, respectively, where higher scores indicate a better mental health (van Krugten et al., 2022). The MHQoL has demonstrated favorable psychometric characteristics, including good internal consistency and construct validity across European populations (van Krugten et al., 2022). These three questionnaires were administered in a standardized manner using identical item wording and response options. To accommodate participant preferences and maximize feasibility, assessments were completed either during a one-to-one in-person session with a researcher or via a secure online questionnaire link. These questionnaires were administered at three time points (see Figure 1). In addition, participants were asked whether and what impactful life events had occurred during the preceding study period. Furthermore, the daily sleep level and daily stress level were measured for 13 weeks throughout the baseline period, the intervention period, and the control period, using two questions delivered automatically each morning via WhatsApp: “How did you sleep last night?” and “How stressed did you feel yesterday?” each scored on a 1–5 scale ranging from very poor to very good for sleep quality and from very calm to very stressed for stress levels. During the follow-up week, participants completed a feedback evaluation to report their product experience.

### 2.6 Statistical analysis

Descriptive statistics for the primary and secondary outcomes were calculated. Cross-over effects between the two conditions and across the three periods were analyzed using two-way repeated measures ANOVA. Normality was evaluated using Shapiro-Wilk tests in combination with visual inspection of the distributions, and Greenhouse-Geisser corrections were applied when the assumption of sphericity was violated. Paired samples *t*-tests were applied to assess the changes in SCI, PSS, and MHQoL scores across periods and conditions, and when assumptions of normality were violated, Wilcoxon Signed Rank Tests were performed. Longitudinal daily sleep level and stress level data were analyzed using multi-level mixed models for repeated measures to assess changes over time. Separate models were fitted for each outcome. A sensitivity analysis using linear regression was conducted on first-period outcomes, with first-period treatment allocation as the independent variable and the corresponding baseline score as a covariate. All analyses were conducted in SPSS version 29, with a significance level of *p* < 0.05 and effect sizes were calculated to provide an indication of the magnitude of the observed effects.

### 2.7 Sample size

*A priori* G*power analysis was conducted to determine the needed sample size to detect an effect on the primary outcome measure (SCI) (Faul et al., 2007). Assuming a two sided *t*-test, an alpha of 0.05, a statistical power of 0.80 and an effect size of d = 0.47 were specified. The expected effect size was based on previously reported SCI outcomes in insomnia research (Espie et al., 2018). The analysis indicated that a total sample size of 36 participants was required. To account for an anticipated dropout rate of 10%, 40 participants in total were recruited.

## 3 Results

### 3.1 Participant flow and baseline characteristics

In total, 88 individuals were assessed for eligibility (Figure 2). Of these, 48 participants provided informed consent and were enrolled. Six participants withdrew before the study initiation and two participants dropped out after. Because recruitment and enrollment occurred sequentially in small participant blocks, an additional block of participants had already started the study procedure before all withdrawals became apparent. This resulted in a final sample of 40 participants who completed the study and were included in the analyses.

**FIGURE 2:**
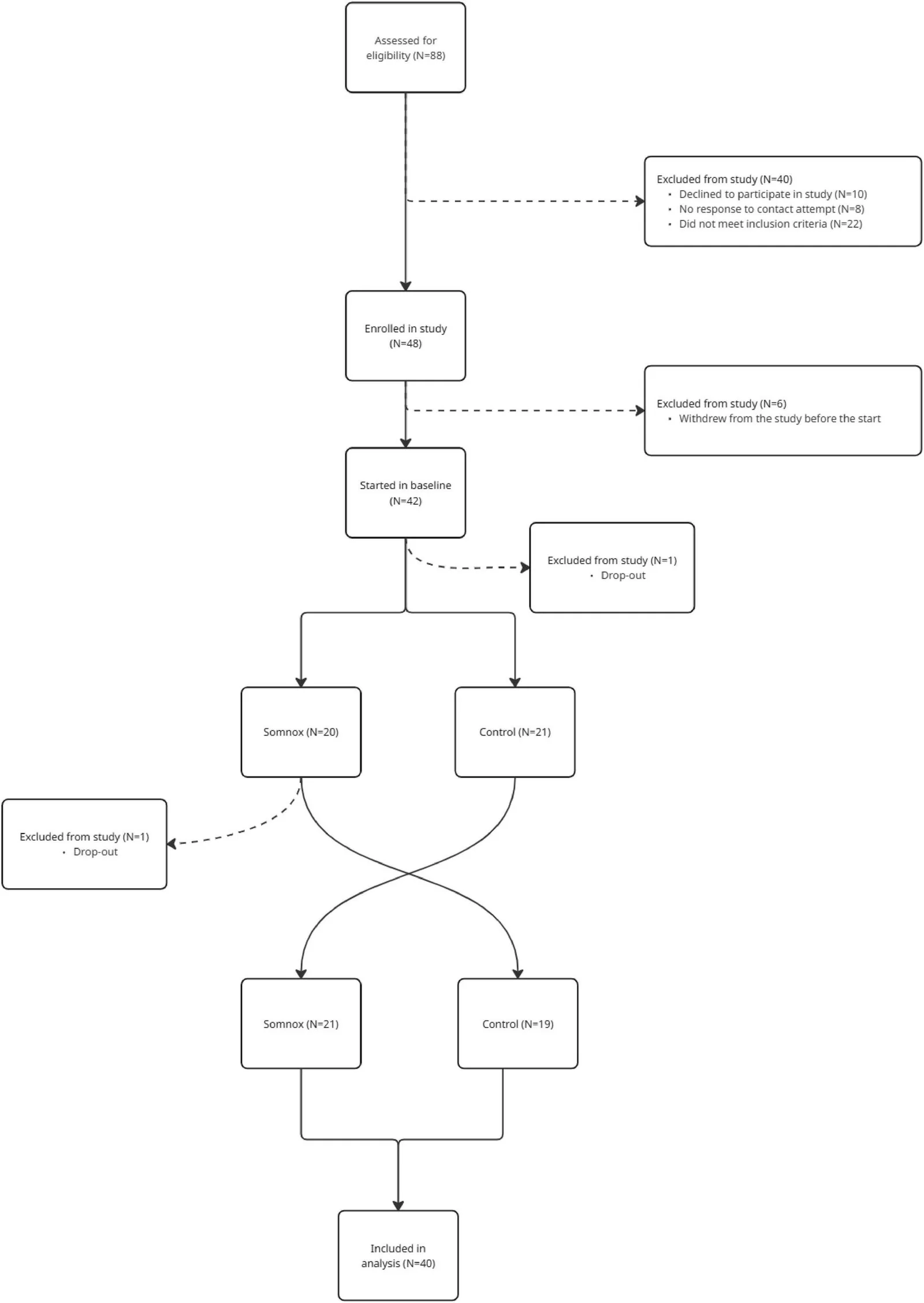
Participant flow chart.

Participants were randomized to one of the two treatment sequences: “Baseline → Somnox → Control” (*N* = 19) or “Baseline → Control → Somnox” (*N* = 21). An overview is provided of the participant characteristics and the distribution across the randomized conditions (Table 1). Overall, the majority of the participants were female (90.0%, *N* = 36). Responses to the open-ended question were categorized into themes representing different types of worries. While most participants reported work-related worries (70.0%, *N* = 28), followed by family (32.5%, *N* = 13) and health worries (20.0%, *N* = 8), the distribution of these characteristics was similar across the two sequences. Some variability between groups was observed, for example in health worries (31.6% vs. 9.5%), relationship worries (5.3% vs. 14.3%), and loneliness worries (0.0% vs. 9.5%), reflecting the relatively small group sizes. Participants could report multiple types of worries.

**TABLE 1:** Participant characteristics and distribution across the randomized conditions.

Participant characteristic	Baseline → Somnox → Control (*N* = 19)	Baseline → Control → Somnox (*N* = 21)	Total (*N* = 40)
Gender (% female)	94.7% (*N* = 18)	85.7% (*N* = 18)	90.0% (*N* = 36)
Financial worries (%)	10.5% (*N* = 2)	9.5% (*N* = 2)	10.0% (*N* = 4)
Health worries (%)	31.6% (*N* = 6)	9.5% (*N* = 2)	20.0% (*N* = 8)
Family worries (%)	31.6% (*N* = 6)	33.3% (*N* = 7)	32.5% (*N* = 13)
Relationship worries (%)	5.3% (*N* = 1)	14.3% (*N* = 3)	10.0% (*N* = 4)
Work pressure worries (%)	63.2% (*N* = 12)	76.2% (*N* = 16)	70.0% (*N* = 28)
Loneliness worries (%)	0.0% (*N* = 0)	9.5% (*N* = 2)	5.0% (*N* = 2)

### 3.2 Sleep quality (SCI)

Overall, the sleep quality improved after the intervention period compared to the baseline period, whereas scores remained relatively stable after the control period compared to the baseline period (Figure 3).

**FIGURE 3:**
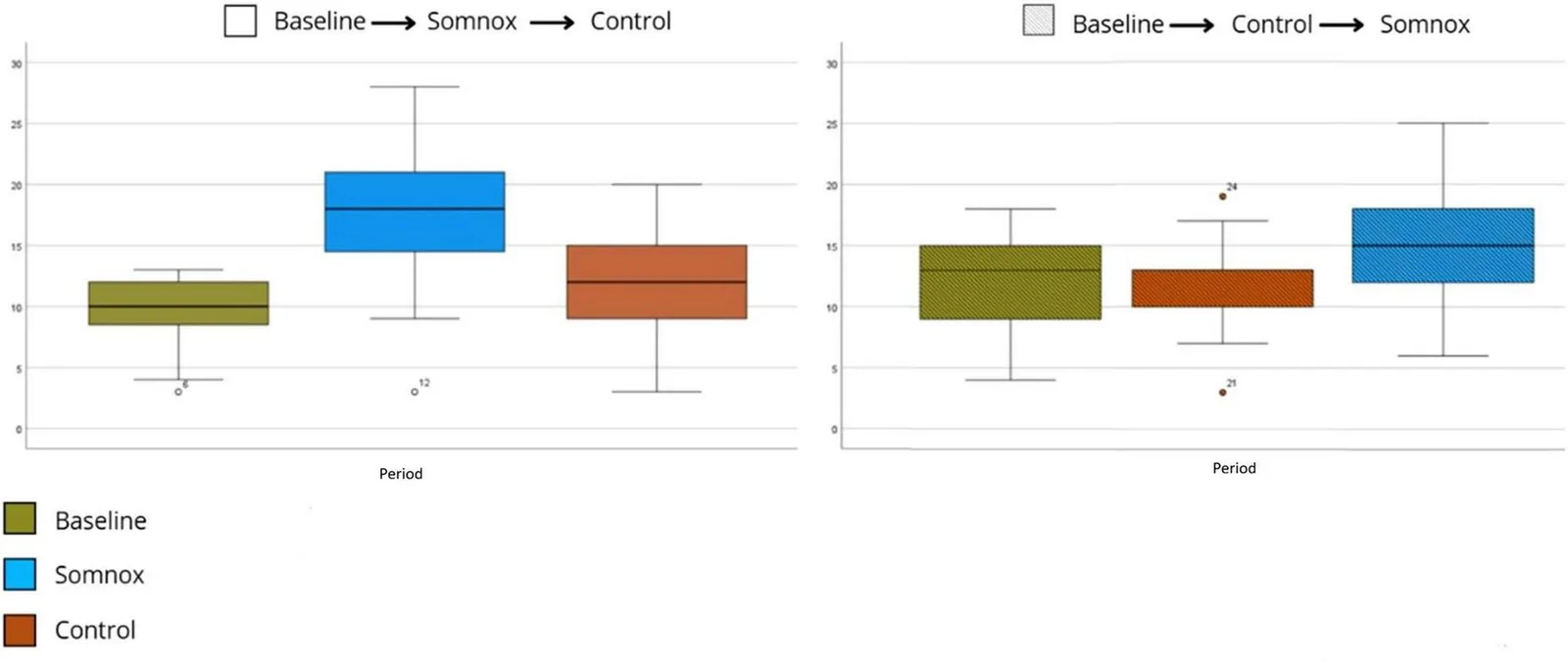
Boxplots showing the Sleep Condition Indicator (SCI) score in every treatment period for each treatment sequence.

The average SCI of participants who first received the Somnox intervention followed by a period without intervention (Baseline → Somnox → Control) increased from 9.74 ± 2.81 after the baseline period to 17.42 ± 6.34 after Somnox use, followed by a decrease to 11.89 ± 4.63 after the control period (Table 2). The average SCI of participants who first had a period without intervention followed by Somnox use (Baseline → Control → Somnox) remained relatively stable from 11.90 ± 3.99 after the baseline period to 12.05 ± 3.56 after the control period, and increased to 15.10 ± 4.96 after Somnox use. The mean within-individual difference between Somnox and Control was 4.22 ± 5.46 (95% CI 2.48 to 5.97, *p* < 0.001).

**TABLE 2:** Mean outcome values for each condition within both treatment sequences, with the associated within-individual difference (Somnox−Control) and the overall treatment effect (SD, standard deviation; CI, confidence interval).

Treatment sequence	Treatment period			Within-individual difference: Somnox−Control
	1	2	3	
Baseline → Somnox → Control				
SCI (mean ± SD)	9.74 ± 2.81	17.42 ± 6.34	11.89 ± 4.63	5.53 ± 6.83**
PSS (mean ± SD)	22.68 ± 5.92	17.68 ± 6.82	18.84 ± 7.30	−1.16 ± 6.22**
MHQoL-7D (mean ± SD)	13.0 ± 3.04	13.58 ± 3.47	13.53 ± 3.39	0.05 ± 2.15
MHQoL-VAS (mean ± SD)	6.42 ± 1.39	6.68 ± 1.38	6.68 ± 1.83	0.00 ± 1.25
Daily sleep level (mean ± SD)	2.71 ± 0.33	3.40 ± 0.40	3.21 ± 0.47	0.20 ± 0.39**
Daily stress level (mean ± SD)	3.21 ± 0.54	2.76 ± 0.46	2.78 ± 0.42	−0.02 ± 0.21**
Sample size	19	19	19	19
Baseline → Control → Somnox				
SCI (mean ± SD)	11.9 ± 3.99	12.05 ± 3.56	15.10 ± 4.96	3.05 ± 3.61**
PSS (mean ± SD)	17.38 ± 4.89	16.67 ± 4.00	14.29 ± 5.81	−2.38 ± 5.07**
MHQoL-7D (mean ± SD)	14.10 ± 2.76	13.86 ± 2.37	14.81 ± 1.99	0.95 ± 1.66
MHQoL-VAS (mean ± SD)	6.57 ± 1.03	6.71 ± 1.01	7.14 ± 0.85	0.43 ± 0.87
Daily sleep level (mean ± SD)	2.73 ± 0.46	2.90 ± 0.40	3.01 ± 0.37	0.11 ± 0.26**
Daily stress level (mean ± SD)	2.95 ± 0.49	2.85 ± 0.44	2.67 ± 0.37	−0.18 ± 0.22**
Sample size	21	21	21	21
Treatment effect				
SCI (mean ± SD, 95% CI)	–	–	–	4.22 ± 5.46**, 2.48 to 5.97
PSS (mean ± SD, 95% CI)	–	–	–	−1.80 ± 5.61*, −3.59 to −0.01
MHQoL-7D (mean ± SD, 95% CI)	–	–	–	0.53 ± 1.94, −0.09 to 1.14
MHQoL-VAS (mean ± SD, 95% CI)	–	–	–	0.23 ± 1.07, −0.12 to 0.57
Daily sleep level (mean ± SD, 95% CI)	–	–	–	0.15 ± 0.33*, 0.05 to 0.26
Daily stress level (mean ± SD, 95% CI)	–	–	–	−0.11 ± 0.23, −0.18 to −0.03
Sample size	–	–	–	40

* and ** indicate statistical significance at *p* < 0.05 and *p* < 0.001, respectively.

The repeated measures ANOVA showed a significant difference between the conditions (*p* < 0.001, partial η^2^ = 0.458) (Table 3). Pairwise comparisons showed significant differences between the intervention period and the control period (*p* < 0.001), between the intervention period and baseline period (*p* < 0.001), and a smaller but significant difference between the control period and the baseline period (*p* = 0.046). In addition, a carryover effect between the intervention and control conditions was found (*p* = 0.016, partial η^2^ = 0.115), indicating that the effect of Somnox use persisted during the control period. No significant group sequence effect was found for SCI (*p* = 0.999, partial η^2^ < 0.001).

**TABLE 3:** Tests of condition effects, carryover effects, and sequence effects for Sleep Condition Indicator (SCI), Perceived Stress Scale (PSS), Mental Health Quality of Life (MHQoL)-7D, and MHQoL-VAS.

Two-way repeated measures ANOVA	Tests of within-subjects effects: Baseline vs. Somnox vs. Control		Tests of between-subjects effects: Somnox → Control or Control → Somnox
	Difference between conditions	Carryover effect between conditions	Group sequence
SCI	*p* < 0.001, η_p_^2^ = 0.458	*p* < 0.05 (*p* = 0.016), η_p_^2^ = 0.115	*p* > 0.05 (*p* = 0.999), η_p_^2^ < 0.001
PSS	*p* < 0.001, η_p_^2^ = 0.231	*p* > 0.05 (*p* = 0.185), η_p_^2^ = 0.043	*p* < 0.05 (*p* = 0.027), η_p_^2^ = 0.123
MHQoL-7D	*p* > 0.05 (*p* = 0.118), η_p_^2^ = 0.055	*p* > 0.05 (*p* = 0.329), η_p_^2^ = 0.029	*p* > 0.05 (*p* = 0.290), η_p_^2^ = 0.029
MHQoL-VAS	*p* < 0.05 (*p* = 0.046), η_p_^2^ = 0.080	*p* > 0.05 (*p* = 0.396), η_p_^2^ = 0.024	*p* > 0.05 (*p* = 0.554), η_p_^2^ = 0.009
**Paired samples *T*-tests**	**% Pairwise comparisons: Baseline vs. Somnox vs. Control**		
	**Difference Somnox−Control**	**Difference Somnox−Baseline**	**Difference Control−Baseline**
SCI	*p* < 0.001	*p* < 0.001	*p* < 0.05 (*p* = 0.046)
PSS	*p* < 0.05 (*p* = 0.049)	*p* < 0.001	*p* < 0.05 (*p* = 0.014)
MHQoL-7D	*p* > 0.05 (*p* = 0.094)	*p* > 0.05 (*p* = 0.06)	*p* > 0.05 (*p* = 0.706)
MHQoL-VAS	*p* > 0.05 (*p* = 0.193)	*p* < 0.05 (*p* = 0.020)	*p* > 0.05 (*p* = 0.160)

At the individual level, 85% of participants showed an increase in SCI after Somnox use, with improvements ranging from 1 to 23 points. Of these, 57.5% showed an improvement of 0–6, while 27.5% improved by 7–23 points. This suggests that over a quarter of participants achieved clinically meaningful change according to the SCI reliable change threshold. Within this subset, 72.7% were participants who first received the Somnox intervention after the baseline period, compared to 27.3% in the other sequence. The most commonly reported type of worries in this subset was work-pressure worries (72.7%), consistent with the overall sample.

### 3.3 Perceived stress (PSS)

Overall, stress decreased after the intervention period compared to the baseline period, whereas scores increased again after the control period (Figure 4).

**FIGURE 4:**
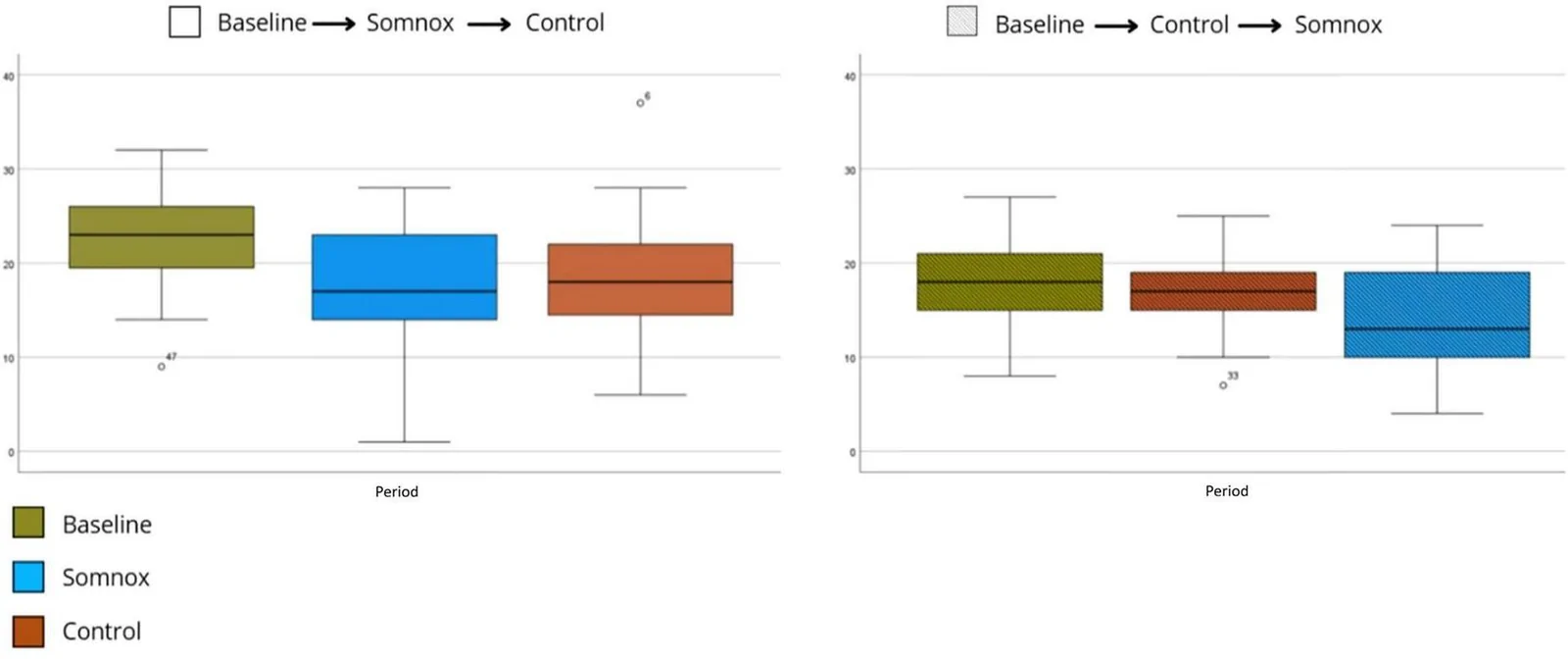
Boxplots showing the Perceived Stress Scale (PSS) score in every treatment period for each treatment sequence.

In the Baseline → Somnox → Control sequence, PSS scores decreased from 22.68 ± 5.92 after the baseline period to 17.68 ± 6.82 after the intervention period, followed by a slight increase to 18.84 ± 7.30 after the control period (Table 2). In the Baseline → Control → Somnox sequence, scores decreased from 17.38 ± 4.89 after the baseline period to 16.67 ± 4.00 after the control period, and further decreased to 14.29 ± 5.81 after Somnox use. The mean within-individual difference between Somnox and Control conditions was −1.80 ± 5.61 (95% CI −3.59 to −0.01, *p* = 0.049).

The repeated measures ANOVA showed a significant difference between the conditions (*p* < 0.001, partial η^2^ = 0.231) (Table 3). Pairwise comparisons showed that PSS scores were significantly lower during the intervention period compared to baseline period (*p* < 0.001) and compared to the control period (*p* = 0.049). A smaller but significant difference was also observed between control period and the baseline period (*p* = 0.014). A significant group sequence effect was found (*p* = 0.027, partial η^2^ = 0.123), indicating that the order in which participants received the conditions influenced overall stress outcomes. No carryover effect was observed, indicating that the effect of Somnox use did not persist into the subsequent condition (*p* = 0.185, partial η^2^ = 0.043).

### 3.4 Quality of life (MHQoL)

For MHQoL outcomes, no clear trends were observed and these stayed equal during all periods and conditions (Table 2).

The repeated measures ANOVA showed no significant differences for MHQoL-7D scores between the periods (*p* = 0.118, partial η^2^ = 0.055), and no sequence effect (*p* = 0.290, partial η^2^ = 0.029) or carryover effect (*p* = 0.329, partial η^2^ = 0.029) was observed (Table 3). The mean within-individual difference between the Somnox and Control conditions was 0.53 ± 1.94 (95% CI −0.09 to 1.14) (Table 2).

For MHQoL-VAS, the repeated measures ANOVA showed a significant difference between the conditions (*p* = 0.046, partial η^2^ = 0.080) (Table 3). Pairwise comparisons indicated that after the intervention period the MHQoL-VAS score was significantly higher than after the baseline period (*p* = 0.020). No significant differences were observed between the intervention period and the control period (*p* = 0.193) or the control period and the baseline period (*p* = 0.160). The mean within-individual difference between the Somnox and Control conditions was non-significant (0.23 ± 1.07; 95% CI −0.12 to 0.57, *p* = 0.193) (Table 2). No carryover effects were observed, indicating that any effect of Somnox use did not persist into the subsequent condition.

### 3.5 Daily sleep level

Overall, daily sleep scores were somewhat higher during the intervention period compared to the baseline and control periods (Figure 5).

**FIGURE 5:**
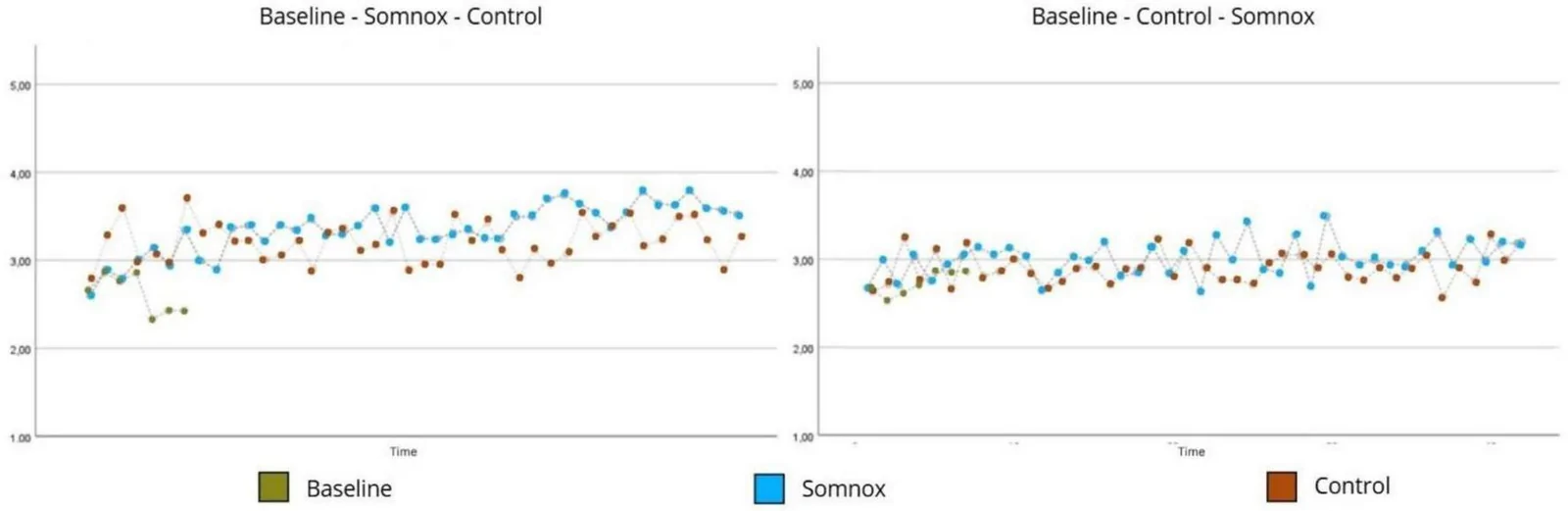
Longitudinal data of the daily sleep level in every treatment period for each treatment sequence.

In the Baseline → Somnox → Control sequence, daily sleep level increased from 2.71 ± 0.33 during the baseline period to 3.40 ± 0.40 during Somnox use, followed by a decrease to 3.21 ± 0.47 during the control period (Table 2). In the Baseline → Control → Somnox sequence, scores increased from 2.73 ± 0.46 during the baseline period to 2.90 ± 0.40 during the control period, and further increased to 3.01 ± 0.37 during Somnox use. The mean within-individual difference between the Somnox and Control conditions was 0.15 ± 0.33 (95% CI 0.05 to 0.26, *p* = 0.012).

The multi-level mixed model showed a significant difference between the conditions (*p* < 0.001) (Table 4). Pairwise comparisons showed that scores during the intervention period were significantly higher than during the baseline period (*p* < 0.001) and also higher than the control period (*p* = 0.012). A carryover effect was observed (*p* < 0.001), indicating that the effect of Somnox use persisted into the subsequent condition. In addition, a significant group sequence effect was found (*p* = 0.011), showing that the order in which participants received the conditions influenced overall daily sleep scores.

**TABLE 4:** Tests of condition effects, carryover effects, and sequence effects for daily sleep and stress levels.

Multi-level mixed model for repeated measures	Tests of fixed effects: Condition: Baseline vs. Somnox vs. Control Group sequence: Somnox → Control or Control → Somnox		
	Difference between conditions	Carryover effect	Group sequence
Daily sleep level	*p* < 0.001	*p* < 0.001	*p* < 0.05 (*p* = 0.011)
Daily stress level	*p* < 0.001	*p* < 0.001	*p* > 0.05 (*p* = 0.70)
**Multi-level mixed model for repeated measures**	**% Pairwise comparisons: Baseline vs. Somnox vs. Control**		
	**Difference Somnox−Control**	**Difference Somnox−Baseline**	**Difference Control−Baseline**
Daily sleep level	*p* < 0.05 (*p* = 0.012)	*p* < 0.001	*p* < 0.001
Daily stress level	*p* > 0.05 (*p* = 0.056)	*p* < 0.001	*p* < 0.001

### 3.6 Daily stress level

Overall, daily stress decreased during the intervention period compared to the baseline period, whereas scores during the control period remained similar to the baseline period (Figure 6).

**FIGURE 6:**
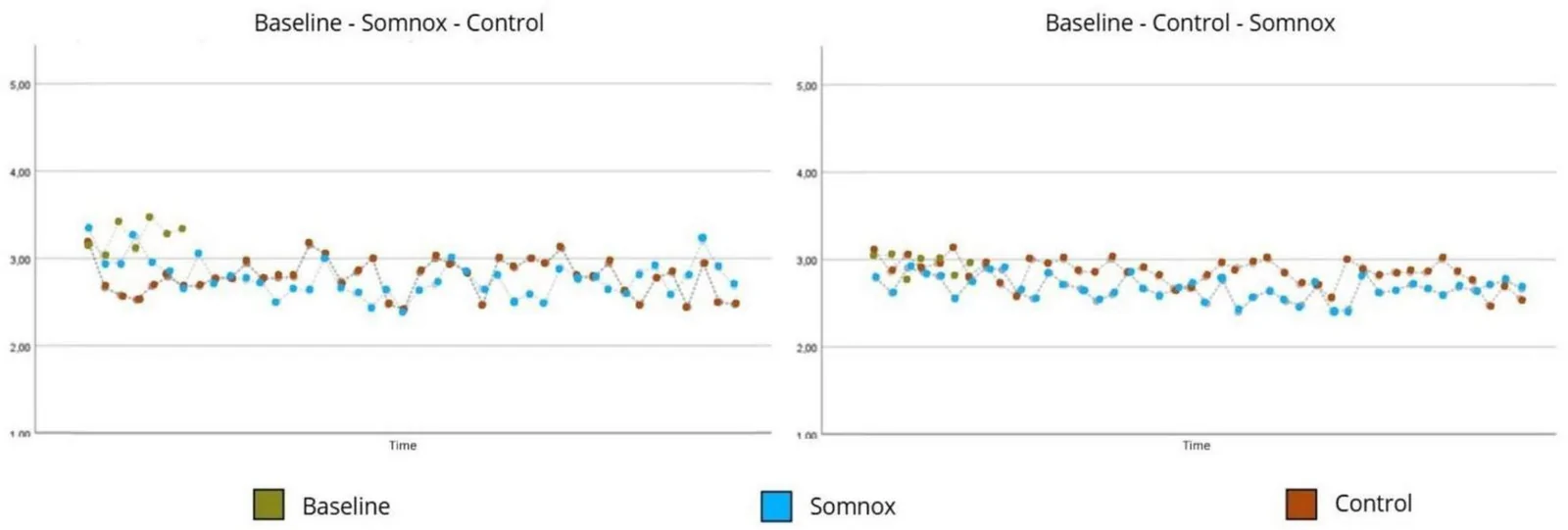
Longitudinal data of the daily stress level in every treatment period for each treatment sequence.

In the Baseline → Somnox → Control sequence, daily stress decreased from 3.21 ± 0.54 during the baseline period to 2.76 ± 0.46 during the intervention period, and remained similar during the control period (2.78 ± 0.42) (Table 2). In the Baseline → Control → Somnox sequence, scores decreased from 2.95 ± 0.49 during the baseline period to 2.85 ± 0.44 during the control period and further decreased to 2.67 ± 0.37 during the intervention period. The mean within-individual difference between the Somnox and Control conditions was −0.11 ± 0.23 (95% CI −0.18 to −0.03, *p* = 0.056).

The multi-level mixed model showed a significant difference between conditions (*p* < 0.001) (Table 4). Pairwise comparisons showed significant decreases from the baseline period to the intervention period (*p* < 0.001) and from the baseline period to the control period, but the difference between the intervention period and the control period did not reach statistical significance (*p* = 0.056). A carryover effect was observed (*p* < 0.001), indicating that the effect of Somnox use persisted into the subsequent condition. No significant group sequence effect was found (*p* = 0.70), suggesting that the order of conditions did not influence overall daily stress scores.

### 3.7 Sensitivity analysis

As a sensitivity analysis, first-period questionnaire outcomes were analyzed using linear regression, comparing participants who received the Somnox intervention during the first treatment period with participants who received the control condition during the first treatment period, prior to crossover. For each outcome, the end-of-period-1 score was included as the dependent variable, first-period treatment allocation as the independent variable, and the corresponding baseline score as a covariate. After adjustment for baseline SCI, participants receiving Somnox during the first period had higher SCI scores than participants receiving the control condition (B = 7.29, 95% CI 4.54 to 10.04, *p* < 0.001) (Table 5). For PSS, the adjusted difference was −2.55 (95% CI −5.58 to 0.47, *p* = 0.095). No statistically significant first-period differences were observed for MHQoL-7D (B = 0.57, 95% CI −0.69 to 1.83, *p* = 0.366) or MHQoL-VAS (B = 0.08, 95% CI −0.44 to 0.60, *p* = 0.757). The first-period analysis therefore supported the primary finding for SCI, with higher sleep quality scores observed in the Somnox group after adjustment for baseline differences and before crossover. The first-period analyses did not provide evidence of differences for PSS, MHQoL-7D, or MHQoL-VAS.

**TABLE 5:** Baseline-adjusted first-period sensitivity analysis.

Outcome parameter	B	95% CI	*P*-value
SCI	7.29	4.54 to 10.04	<0.001
PSS	−2.55	−5.58 to 0.47	0.095
MHQoL-7D	0.57	−0.69 to 1.83	0.366
MHQoL-VAS	0.08	−0.44 to 0.60	0.757

B represents the baseline-adjusted difference between participants receiving Somnox and control during the first treatment period. Positive values indicate higher scores in the Somnox group; negative values indicate lower scores.

### 3.8 Impactful life events and feedback evaluation

Each participant reported at the three assessment points whether any impactful life events had occurred during the preceding study period. Reported events covered a wide range of personal, family, health-related, and work-related experiences. Examples of health-related events were serious illness, hospitalizations or medical diagnoses. Family and relationship-related events were also frequently reported, such as relationship conflicts or break-ups, complex family dynamics, caregiving responsibilities for ill relatives, and situations involving children or partners. Examples of work-related experiences were high workload, burnout, conflicts with colleagues, or challenges during reintegration after illness. Examples of personal life developments were starting therapy, setting personal boundaries, ending relationships that caused stress, or learning to stand up for themselves. Some events like pregnancy, the birth of family members or ending a difficult relationship that could be challenging but also lead to greater emotional relief, were described as having both positive and negative impact on their life, depending on the circumstances. Other reported experiences included vacations, receiving recognition at work, finding a new job, and periods of personal growth. A visual inspection of the reported events across the three measurement periods suggested that impactful life events occurred throughout the study did not show clear clustering within a specific treatment period for individual participants.

During the follow-up evaluation, participants provided feedback regarding their experiences with the Somnox. Participants reported adherence to the instruction to use the Somnox device nightly during the intervention period The device received an average rating of 7.3 out of 10. Furthermore, 75% of participants rated the Somnox as a high-quality or very high-quality product. All participants indicated that they had used the Somnox according to the instructions provided.

## 4 Discussion

This crossover randomized controlled trial evaluated the effectiveness of the Somnox as an assistive sleep technology to support adults experiencing poor sleep quality and high stress due to worrying. The primary objective was to determine whether Somnox use improves perceived sleep quality and reduces perceived stress, while the secondary outcome included quality of life across a 13-week period. Previous research on the Somnox 2 demonstrated mixed effects on sleep and stress-related outcomes. In a randomized controlled trial among adults with insomnia, Støre et al. (2023) found that a 3-week intervention with the Somnox did not lead to statistically significant improvements in insomnia severity. In a randomized controlled feasibility study in individuals with posttraumatic stress disorder, Lotzin et al. (2025) reported that while the intervention was feasibly and well-accepted, and led to significantly greater reductions in distress compared to a control condition, no significant improvements were observed in sleep quality or wellbeing. In line with previous findings, the present study also observed reductions in perceived stress. However, in contrast to earlier research, this crossover randomized controlled trial found consistent improvements in perceived sleep quality across conditions. These findings provide additional empirical evidence on the potential effectiveness of the Somnox. No clear evidence was found for improvements in quality of life.

### 4.1 Main findings of the outcome measurements

The main results showed consistent improvements in SCI after Somnox use compared to both the baseline period and the control period. Across both treatment sequences, SCI increased after the intervention period and decreased after the control period, suggesting a clear within-participant effect. The estimated overall treatment effect (Somnox – Control) of 4.22 points reflects a meaningful significant improvement in sleep quality. In addition, most participants (85%) showed individual improvements in SCI after Somnox use.

At the same time, the distinction between statistical significance and clinical significance should be considered when interpreting these findings. SCI improvements varied widely per participant (1–23 points) and only a subset of 27.5% of participants improved by at least seven points, which is considered reliable clinical change according to SCI reference criteria. The individual differences in SCI improvements may reflect variations in how participants used the Somnox within the provided instructions, suggesting a potential dose-response relationship between device usage and sleep quality outcomes. The fact that the mean improvement in SCI remained below the threshold considered indicative of reliable clinical change shows that the Somnox use can achieve reliable clinical change only to a subset of participants. However, the present study was not designed to determine which specific participant characteristics were associated with clinically meaningful improvement. In this study, participants subjectively reported improvements in sleep quality, which was the primary focus of the study. Future research with larger and diverse samples could examine the subgroup associated with clinically meaningful improvements in sleep outcomes in more detail to better understand the specific participant characteristics associated with the larger improvements in SCI.

Perceived stress (PSS) decreased after Somnox use, with significant differences observed for both treatments sequences between the intervention period and both the baseline and control periods. This pattern supports the hypothesized stress-reducing potential of Somnox.

No clear trends or improvements were observed for quality of life (MHQoL), what may be expected given the short intervention period and the multifactorial determinants of quality of life. Quality of life may require longer intervention periods, or more comprehensive interventions targeting multiple life domains.

Daily measures largely matched the results from the questionnaire outcomes (SCI and PSS). The daily sleep level was significantly higher during the intervention period compared to the control period, supporting a consistent effect on subjective sleep experience across time. Daily stress ratings were lower during Somnox use, but the difference between the intervention and control conditions showed no statistical significance (*p* = 0.056). This may indicate that daily stress is more sensitive to contextual fluctuations (e.g., daily events, workload, family factors) than sleep ratings, or that the effect size is smaller and requires a larger sample to detect effects reliably. This also shows a strength of this study; the inclusion of both validated questionnaires and daily longitudinal measures strengthens the interpretation of trends over time.

### 4.2 Study design considerations

This study illustrates the interplay between carryover effects, period effects, and group sequence effects, which are inherent to a crossover design. Significant interaction effects between conditions and treatment sequence indicated the presence of carryover effects for SCI and daily sleep and stress outcomes. This suggests that the effects of Somnox use may persist beyond the intervention period and influence subsequent measurements. While such residual effects may reflect a beneficial continuation of treatment impact (e.g., increased relaxation or improved sleep-related awareness), they also complicate the interpretation of differences between conditions.

To further explore the potential influence of carryover effects, a sensitivity analysis using only first-period data was conducted. After adjustment for baseline differences, SCI scores were significantly higher in participants receiving Somnox during the first treatment period compared with participants receiving the control condition. This first-period finding supports the primary crossover finding for SCI and was observed before any potential post-crossover carryover could have occurred. In contrast, the first-period analyses did not provide evidence of differences for PSS, MHQoL-7D, or MHQoL-VAS. These findings should nevertheless be interpreted cautiously, as the first-period analyses involve smaller between-group comparisons and therefore do not retain the within-subject advantage of the crossover design. Moreover, significant carryover effects were detected in the primary crossover analyses, indicating that the 1-day washout period was likely insufficient to fully eliminate residual intervention effects.

This phenomenon is further observed by the several significant differences between the control and baseline periods, despite the absence of an intervention during both these periods. These differences may reflect natural fluctuations over time, seasonal or situational influences, or behavioral changes initiated during the study, such as improved sleep hygiene or increased awareness of stress and relaxation. In addition, significant group sequence effects for perceived stress and daily sleep outcomes indicate that the order in which participants received the intervention influenced the results. This may be due to expectancy effects, learning effects, or differences in contextual factors such as impactful life events.

Together, these findings indicate that treatment effects in this study cannot be interpreted independently from temporal and sequence-related influences. Although the crossover design is a strength in terms of within-participant comparison and statistical efficiency, the 1-day washout period was likely insufficient to fully eliminate residual intervention effects and may therefore have limited full separation between intervention periods. Future studies may benefit from a longer washout period or a parallel-group design to better isolate the treatment effect and reduce potential contamination between conditions.

### 4.3 Impactful life events

Participants reported impactful life events throughout the study. Such events are highly relevant in this population, as worries and stressors are central to the insomnia-stress cycle. No clear clustering of such events across treatment periods was observed in a visual inspection. However, these events were assessed qualitatively and in a relatively general way. Future research into these impactful experiences could benefit from more systematic or quantitative assessment to better account for their potential influence on sleep and stress outcomes.

### 4.4 Other limitations and possible future research

The sample consisted predominantly of female participants, limiting generalizability. Additionally, participants were recruited from different contexts (partner organizations, university, hospital staff), and variation in type of worries (e.g., work pressure vs. financial or health worries) may have influenced responsiveness to the intervention.

Evaluating longer-term effects beyond six weeks and investigating changes after discontinuation could give more insight into whether the Somnox contributes to lasting improvements in sleep quality and stress level.

The present study focused on subjective self-reported outcomes, as the primary aim was to evaluate participants’ own experiences with sleep, stress and quality of life. Subjective experiences are considered highly relevant in the context of insomnia and stress-related complaints. At the same time, objective sleep measurements such as actigraphy or polysomnography may provide additional insights into physiological sleep outcomes. These measures were not included in the current study because they fell outside the primary aim and would have increased the complexity and participant burden of the study. Future research may benefit from combining subjective and objective sleep measures.

The intervention in this study involved active use of the Somnox 2 as a complete intervention and focused on participants’ subjective experiences of sleep and stress. The aim was to evaluate the potential value of using the device under conditions resembling its intended use, rather than to isolate the specific effects of respiration-contingent breathing guidance. Therefore, the no-intervention control condition was considered appropriate for the aim of the present study. However, this design does not allow the effects of the Somnox 2 to be fully distinguished from potential placebo, expectancy, or other non-specific effects associated with using the device and increased awareness of sleep. Therefore, the observed improvements should be interpreted cautiously. Future research using a sham-controlled design could help isolate the specific contribution of the breathing-based intervention.

No objective device-use data, such as exact usage duration or detailed interaction logs, were collected in the present study, in order to reduce participant burden and minimize the collection of privacy-sensitive data. As a result, it remains unclear whether differences in frequency or duration of Somnox use were associated with the observed outcomes. Future research may further investigate the relationship between usage patterns and intervention effectiveness by including more detailed adherence measures.

## 5 Conclusion

This crossover randomized controlled trial demonstrated that Somnox use significantly improved perceived sleep quality and reduced perceived stress in adults experiencing sleep problems and high stress due to worrying. No clear effect was found on quality of life outcomes. Overall, Somnox appears to be a feasible and promising supportive sleep technology for individuals experiencing stress-related sleep problems.

## Data Availability

The datasets presented in this article are not readily available because of privacy and ethical restrictions. Requests to access the datasets should be directed to h.s.brummer@hhs.nl.
